# Cannabis‐Derived Compounds Against *Plasmodium* sp.: A Systematic Review of Preclinical Studies

**DOI:** 10.1111/tmi.70044

**Published:** 2025-10-15

**Authors:** Tácio de Mendonça Lima, Luann Wendel Pereira de Sena, Ana Carolina Corrêa de Sousa, Gabriel Rodrigues Martins de Freitas, Inajara Rotta, Marília Berlofa Visacri

**Affiliations:** ^1^ Department of Pharmacy and Pharmaceutical Administration Faculty of Pharmacy, Fluminense Federal University Niteroi Brazil; ^2^ Faculty of Collective Health Federal University of the South and Southeast of Para Marabá Brazil; ^3^ Faculty of Pharmacy Federal University of Rio de Janeiro Rio de Janeiro Brazil; ^4^ Department of Pharmaceutical Sciences Federal University of Paraiba Joao Pessoa Brazil; ^5^ Department of Pharmacy Federal University of Parana Curitiba Brazil; ^6^ Department of Pharmacy, Faculty of Pharmaceutical Sciences University of Sao Paulo Sao Paulo Brazil

**Keywords:** cannabis, malaria, plasmodium, preclinical drug evaluation

## Abstract

**Objective:**

This study aims to evaluate preclinical studies on the effects and toxicity of cannabis‐derived compounds against *Plasmodium* sp.

**Methods:**

A literature search was conducted in Web of Science, PubMed, Scopus and LILACS databases until December 2024. Studies that assessed the activity or toxicity of cannabis against *Plasmodium* sp. in in vitro or in vivo studies were included. Two reviewers independently performed the study selection, data extraction and methodological assessment.

**Results:**

Eight studies published between 2001 and 2022 were included, with the majority conducted in North America (*n* = 5). Most in vitro studies focused on assessing antimalarial activity through half‐maximal inhibitory concentration (IC_50_), which ranged from 0.16 to 4.1 μg/mL, indicating mild to high activity. For the in vivo studies, all reported positive effects, including moderate antimalarial activity and disease tolerance. The toxicity profile of these compounds has not been extensively studied, and most studies present an unknown or unclear risk of bias due to insufficient methodological information.

**Conclusions:**

Future studies should provide more comprehensive details on study design and further validate these findings, especially concerning toxicity.

## 1 Introduction

Malaria remains one of the most significant parasitic diseases worldwide, contributing to substantial morbidity and mortality, particularly in tropical and subtropical regions [1]. Recognised as a neglected disease, *Plasmodium* sp. infection is transmitted by mosquitoes of the *Anopheles* genus, with *Plasmodium falciparum* being the most lethal species and responsible for most malaria‐related deaths [2]. In 2022, the World Health Organization (WHO) estimated approximately 249 million cases and 608,000 malaria‐related deaths across 85 endemic countries, with Nigeria, the Democratic Republic of the Congo, Uganda and Mozambique accounting for nearly half of all cases [1].

Although *P. falciparum* predominates in the development of malaria, other *Plasmodium* species also represent significant risks. For example, *Plasmodium malariae* has been linked to severe complications, including severe anaemia, pulmonary issues and kidney failure [3]. A systematic review by Kotepui et al. [4] found that approximately 3% of patients infected with *P. malariae* developed severe forms of the disease, with an estimated mortality rate of 0.17%. These findings emphasise the need for therapeutic alternatives targeting multiple *Plasmodium* species and help reduce the overall disease burden.

Advances in malaria control include insecticides, chemoprophylaxis and the development of vaccines such as RTS,S/AS01 and R21/Matrix‐M. However, these measures have limited efficacy, and the emergence of strains resistant to conventional therapies highlights the need for new therapeutic approaches [1, 5]. Currently, antimalarial treatments predominantly rely on artemisinin‐based combination therapies (ACTs), which, although effective, are increasingly challenged by the growing resistance of *P. falciparum* [2, 6, 7].

In this context, compounds derived from medicinal plants, particularly from species found in Africa, Asia and South America, have been explored for their potential antimalarial properties [8]. *Cannabis sativa* has been extensively studied for its therapeutic options in various conditions, including inflammatory [9, 10] and neurological diseases [10, 11]. Previous studies have described the potential effects of cannabis on malaria vector [12] and antimalarial activity [13, 14, 15]. These findings suggest that cannabis‐based products could provide an alternative or complementary therapeutic approach to conventional treatments, particularly in light of the increasing resistance to existing drugs.

Given the increasing interest in using cannabis and derivatives for malaria treatment, this systematic review aims to evaluate preclinical studies that examine the effects or toxicity of cannabis‐derived compounds against *Plasmodium* sp. The findings of this review are expected to enhance understanding of the therapeutic potential of these compounds in malaria control and support the design of future translational studies and clinical trials.

## 2 Materials and Methods

This systematic review followed the Preferred Reporting Items for Systematic Reviews and Meta‐Analyses Statement (PRISMA) 2020 checklist and reporting guideline [16]. The protocol was registered on the International Prospective Register of Systematic Reviews (PROSPERO; registration number CRD42023423643).

### 2.1 Literature Databases and Search Strategy

A comprehensive literature search was conducted to identify relevant studies published from the inception of the database until December 31st, 2024, in the Web of Science, PubMed, Scopus and Latin American and Caribbean Health Sciences Literature (LILACS) databases. The search strategy included keywords and medical subject headings related to ‘Cannabis’ and ‘Plasmodium’. In addition, a grey literature search in Google Scholar of up to 60 registers was conducted, excluding patents and citations, to identify non‐indexed studies in the databases used. The reference lists of studies included were searched. The full search strategies for all databases can be found in Data S1.

### 2.2 Eligibility Criteria

Studies that assessed the activity or toxicity of cannabis‐derived compounds against *Plasmodium* sp. in preclinical studies (in vitro or in vivo) were included. Studies written in non‐Roman characters (e.g., Japanese, Chinese, Russian), performed in humans, that did not report the activity or toxicity of cannabis‐derived compounds, in silico studies and conducted with other pathogens were excluded. Reviews, letters to the editor, conference proceedings and other non‐peer‐reviewed documents were also excluded.

### 2.3 Study Selection

The studies retrieved from the databases were allocated to the Rayyan QCRI web platform [17] for screening. The process involved three steps: (1) removing duplicates, (2) analyzing titles and abstracts and (3) reviewing the full texts of studies.

All registers were independently screened and selected by two reviewers (L.W.P.S. and A.C.C.S.), and any disagreement was resolved by the third investigator (T.M.L.). When the full texts were unavailable in the databases, the corresponding authors were contacted via email or through the Researchgate platform (www.researchgate.net).

### 2.4 Data Extraction

Information was extracted based on the study design. For in vitro studies, the collected data included authors, year of publication, country, compounds, cell type, assays related to antimalarial activity, main findings, study limitations and funding. For in vivo studies, the extracted data encompassed authors, year of publication, country, animal, intervention, comparator, outcome measures, main findings, limitations and funding.

Two authors (L.W.P.S. and A.C.C.S.) independently completed the data extraction, using a preformatted spreadsheet in Microsoft Excel. Disagreements were resolved by discussion with the third author (T.M.L.).

### 2.5 Quality Assessment

The studies' methodological quality was assessed using Golbach's tool [18] for in vitro studies and SYRCLE's risk of bias [19] for in vivo studies. The Golbach's tool comprises eight items across four domains: (1) Performance bias, (2) Selection bias, (3) Detection bias and (4) Other bias. The SYRCLE's risk of bias criteria encompass 10 items across six domains: (1) Selection bias, (2) Performance bias, (3) Detection bias, (4) Attrition bias, (5) Reporting bias and (6) Other bias. Each item was rated as ‘low risk’, ‘high risk’ and ‘unclear risk’. Two independent reviewers (I.R. and T.M.L.) assessed the studies, and any discrepancies were resolved by consensus.

### 2.6 Data Synthesis

The characteristics of the included studies were summarised descriptively through a narrative synthesis and structured tables. The original ideas and concepts presented in the included studies were acknowledged and preserved. A meta‐analysis was not planned due to the expected heterogeneity among the studies.

## 3 Results

### 3.1 Search Results

**FIGURE 1:**
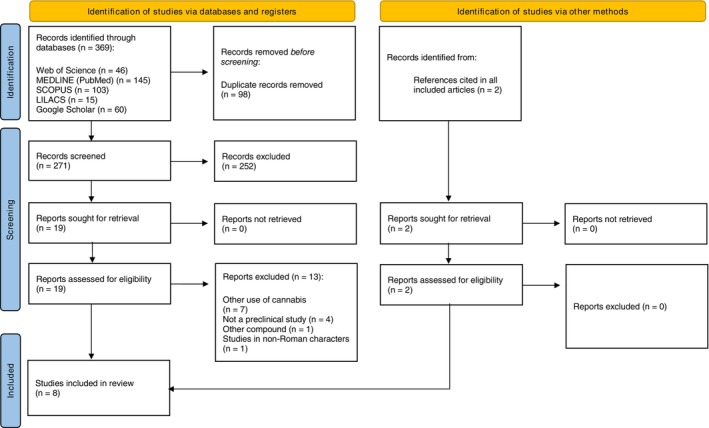
Study selection flowchart through literature search.

The electronic search found 369 potential registers. After removing duplicates and reviewing the titles and abstracts, 19 articles were selected for full‐text reading. In addition, two studies were identified through references cited by these articles. Of these, eight [20, 21, 22, 23, 24, 25, 26, 27] studies 20–27 met the inclusion criteria and were included for review. A flowchart of the literature search is shown in Figure 1.

### 3.2 Characteristics of the Included Studies

Eight studies included in this review were published between 2008 and 2022, of which five were classified as in vitro studies [20, 21, 22, 23, 24] and three were in vivo studies [25, 26, 27]. The majority were performed in North America (*n* = 4) [20, 21, 23, 24], followed by Africa (*n* = 3) [22, 25, 27] and South America (*n* = 1) [26].

Regarding the in vitro studies, most (*n* = 4) [20, 21, 22, 23] tested cannabinoid compounds and derivatives, while one study focused on non‐cannabinoid compounds derived from *C. sativa* [24]. The majority of studies used *P. falciparum* D6 and W2 strains [20, 21, 23, 24] and all assessed antimalarial activity through cultured *P. falciparum* cells using the half‐maximal inhibitory concentration (IC_50_) parameter. One study also performed the β‐haematin test [22]. Four studies [20, 21, 23, 24] identified active compounds with mild antimalarial activity for *P. falciparum* D6 and W2 cell types, with IC_50_ values ranging from 0.90 to 4.76 μg/mL. Only one study [23] reported high antimalarial activity with IC_50_ of 0.16 μg/mL and 0.20 μg/mL for D6 and W2 clone cell types, respectively. Sousa et al. described that the cannabidiol (CBD) compound exhibited mild antimalarial activity (IC_50_ value of 4.1 μg/mL) against chloroquine‐sensitive strains (*Pf*NF54). On the other hand, the delta‐9‐tetrahydrocannabinol (THC) compound exhibited high antimalarial activity with IC_50_ of 0.79 μg/mL and 0.72 μg/mL for *Pf*NF54 and *Pf*K1 cell types, respectively. Regarding the β‐haematin test, THC and CBD exhibited IC_50_ values of 11.3 and 51.0 μM, respectively, for β‐haematin inhibition. The studies highlighted limitations in their findings, including a limited number of compounds tested [21, 23] and the inherent psychoactive effects of THC that may impair its antimalarial effects [22]. Two studies [21, 24] did not report any limitations. All the studies reported having received funding sources.

For the in vivo studies, two studies used Swiss albino mice infected with *P. berghei* [25] or *P. falciparum* [27], and one study employed C57BL/6 mice infected with *P. berghei* [26]. Cannabis extracts were used as an intervention in two studies [25, 27], while isolated CBD was used in another study [26]. Chloroquine was used as a comparator in two studies [25, 27], whereas artesunate was used in another study [26]. Several outcomes of interest were investigated, including parasitemia, survival, haematological and histological analysis, behavioural assessment and cytokine levels. All studies reported positive effects of drugs, including moderate antimalarial activity and disease tolerance, cognitive function improvement, neuroprotective effects, and reduction of pro‐inflammatory cytokines [25, 26]. Additionally, a reduction in parasitemia and an improvement in red blood cells, platelets and haematocrit levels were observed [27]. One study [26] did not report the limitations of the findings. Two studies did not report having received funding sources.

Tables 1 and 2 summarize the key characteristics of the studies included in this systematic review.

**TABLE 1:** Characteristics of in vitro studies included in the systematic review.

Author, year	Country	Compounds	Cell type and assays related to antimalarial activity	Main findings	Study limitation	Funding
Ahmed et al. 2015 [20]	United States	Nine oxygenated cannabinoids of *C. sativa* L. variety	*P. falciparum* D6 clone (CQ‐sensitive) and *P. falciparum* W2 clone (CQ‐resistant) cultures	Compound 9 showed mild antimalarial activity against *P. falciparum* D6 clone (CQ‐sensitive) and *P. falciparum* W2 clone (CQ‐resistant) with IC_50_ values of 3.4 and 2.3 μg/mL, respectively.	NR	National Center for Research Resources and National Institute on Drug Abuse
Ahmed et al. 2022 [21]	United States	Twelve *C. sativa* ‐derived CBD metabolites from 3 microorganisms	*P. falciparum* D6 clone (CQ‐sensitive) and *P. falciparum* W2 clone (CQ‐resistant) cultures	Metabolite 11 (11.5 mg, 3.83% yield by *Absidia glauca*) showed mild antimalarial activity against *P. falciparum* D6 clone (CQ‐sensitive) and *P. falciparum* W2 clone (CQ‐resistant) with IC_50_ values of 2.2 and 2.5 μg/mL, respectively.	The study tested 31 microorganisms, of which only 3 were capable of CBD in their systems, indicating a limited biotransformation capacity among the tested microorganisms.	National Institute on Drug Abuse
de Sousa et al. 2021 [22]	South Africa	CBD and THC of Cannabis spp.	*P. falciparum Pf*NF54 (CQ‐sensitive) and *Pf*K1 (CQ‐resistant) cultures and the β‐haematin test	CBD showed mild antimalarial activity against *P. falciparum* PfNF54 (CQ‐sensitive) with an IC_50_ value of 4.1 μg/mL and an IC_50_ value of 51.1 μM for the β‐haematin test. THC showed high antimalarial activity against *P. falciparum* PfNF54 (CQ‐sensitive) and *P. falciparum* PfK1 (CQ‐resistant) with IC_50_ values of 0.79 and 0.72 μg/mL, respectively. THC showed an IC_50_ value of 11.3 μM for the β‐haematin test.	The psychoactive effects of THC are an undesirable limitation that need to be overcome to optimise the antimalarial effects.	National Research Foundation of South Africa
Osman et al. 2018 [23]	United States	Eight bioactive products from singlet oxygen photooxygenation of Δ9‐THC, Δ8‐THC, Δ9‐THCA, and derivatives, isolated from *C. sativa*	*P. falciparum* D6 clone (CQ‐sensitive) and *P. falciparum* W2 clone (CQ‐resistant) cultures	Compound 14 showed high antimalarial activity against *P. falciparum* D6 clone (CQ‐sensitive) and *P. falciparum* W2 clone (CQ‐resistant) with IC_50_ values of 0.16 and 0.20 μg/mL, respectively. Compounds 9, 11, 20, 25, 28, 30 and 31 presented mild antimalarial activity against *P. falciparum* D6 clone (CQ‐sensitive) and *P. falciparum* W2 clone (CQ‐resistant) with IC_50_ values ranging from 1.0 to 4.76 μg/mL and from 0.90 to 4.5 μg/mL, respectively.	The photooxygenation of Δ8‐THC generated compounds 13 and 14. However, Δ8‐THC and compound 13 were not tested.	National Institute on drug Abuse and the United States Department of Agriculture
Radwan et al. 2008 [24]	United States	Non‐cannabinoid compounds isolated from *C. sativa* L. variety	*P. falciparum* D6 clone (CQ‐sensitive) and *P. falciparum* W2 clone (CQ‐resistant) cultures	Compound 1 (5‐acetoxy‐6‐geranyl‐3‐n‐pentyl‐1,4‐benzoquinone) showed mild antimalarial activity against *P. falciparum* D6 clone (CQ‐sensitive) and *P. falciparum* W2 clone (CQ‐resistant) with IC_50_ values of 2.8 and 2.6 μg/mL, respectively. Compound 9 (6‐prenyl apigenin) showed mild antimalarial activity against *P. falciparum* D6 clone (CQ‐sensitive) and *P. falciparum* W2 clone (CQ‐resistant) with IC_50_ values of 2.8 and 2.0 μg/mL, respectively.	NR	National Center for Research Resources and National Institute on Drug Abuse

Abbreviations: CBD, cannabidiol; CQ, chloroquine; IC_50_, half‐maximal inhibitory concentration; NMR, nuclear magnetic resonance; pLDH, plasmodium lactate dehydrogenase; THC, tetrahydrocannabinol.

**TABLE 2:** Characteristics of in vivo studies included in the systematic review.

Author, year	Country	Animal	Intervention	Control	Outcome measures	Main findings	Study limitations	Funding
Akinola et al. 2018 [25]	Nigeria	Swiss albino mice infected with CQ‐resistant *P. berghei* ANKA	Oral cannabis diet formulations (40%, 20%, 10% and 1%) prepared from dried leaves, twigs, and seeds of the *C. sativa ad libitum* for 14 days.	Positive control: CQ 10 mg/kg/day for 3 days. Negative control: water.	Intrinsic antimalarial activity, survival rate, haematological analysis and histological examination.	No significant difference (*p* > 0.05) in day‐4 parasitemia suppression was observed between the IGs fed with 1%, 10% and 20% formulations and the negative CG. However, a significant increase in day‐4 parasitemia suppression was observed in the IG fed with the 40% formulation (*p* = 0.001). The mean survival time was similar only between the GI‐fed 40% formulation and the positive CG. No statistically significant differences (*p* > 0.05) were found in the haematological indices between all IGs and the negative CG. Histological analysis of the groups revealed no morphological alterations in the panoramic presentation of the prefrontal cortex and hippocampal layers.	The use of dried whole cannabis plant rather than cannabis inflorescence may have limited the antimalarial activity observed. The oral ingestion route may have limited the release of certain antimalarial constituents like terpenoids. Variability in cannabis constituents between cultivars may produce different results. The study focused on whole cannabis consumption rather than isolated constituents, which may be more therapeutically validated.	None
Campos et al. 2015 [26]	Brazil	Female C57BL/6 mice (6–8 weeks old) infected with *P. berghei* ANKA	CBD (30 mg/kg/day, administered intraperitoneally for 3 or 7 days) alone or with Artesunate 64 mg/kg/day for one day and 32 mg/kg/day for 4 days.	Placebo and Artesunate 64 mg/kg/day for one day and 32 mg/kg/day for 4 days.	Parasitemia assessment, behavioral analysis, levels of proinflammatory cytokines (TNF‐α and IL‐6) in the hippocampus and prefrontal cortex, and levels of the neurotrophin BDNF in the hippocampus.	CBD significantly improved survival rates (*p* < 0.001) without affecting parasitemia, compared to the placebo. The Artesunate + CBD treatment resulted in higher survival rates and more sustained parasite clearance compared to Artesunate alone. The Artesunate + CBD treatment had a complete rescue of the clinical signs of Cerebral Malaria. Artesunate + CBD treatment fully restores the cognitive performance of infected animals (*p* < 0.05). The anxiogenic‐like effect was prevented by CBD and Artesunate + CBD treatments. CBD + Artesunate treatment significantly increased BDNF expression when compared to all other groups (*p* < 0.001). CBD + Artesunate treatment reduced the proinflammatory cytokine levels, specifically TNF‐α in the hippocampus (*p* < 0.01) and IL‐6 in the prefrontal cortex (*p* < 0.05).	NR	National Council for Scientific and Technological Development and the Research Support Foundation of the State of Minas Gerais
Nwonuma et al. 2022 [27]	Nigeria	Swiss albino mice infected with *P. berghi* NK‐65 (CQ‐sensitive)	Ethanolic cannabis leaf extract at doses of 100, 200, and 400 mg/kg/day for 4 days	CQ 10 mg/kg/day for 4 days	Parasitemia and haematological analysis	The IG exhibited a significant reduction (*p* < 0.05) in percentage parasitemia and an increase in percentage inhibition compared to the CG. RBC count, platelet count, haematocrit, and percentage weight gain showed a significant increase (*p* ≤ 0.05) in the IG compared to the CG.	Small sample size (5 mice per group). Potential lack of random assignment to infected and non‐infected groups.	NR

Abbreviations: BDNF, brain‐derived neurotrophic factor; CBD, cannabidiol; CG, control group; CQ, chloroquine; IG, intervention group; IL‐6, interleukin‐6; RBC, red blood cells; TNF‐α Tumour necrosis factor‐alpha.

### 3.3 Quality Assessment

Tables 3 and 4 show the methodological quality of the included studies. Overall, most items were classified as ‘unknown bias’ in in vitro studies. For item 2 (‘Is the temperature controlled?’), four studies [20, 21, 23, 24] were classified as ‘moderate bias’. Ahmed et al. [21] classified ‘low bias’ for item 6 (‘Were the methods the same for control and exposure treatment?’), while all studies considered ‘low bias’ for item 8 (‘Was there no industry sponsorship involved?’). Regarding the in vivo studies, there was greater variability in the risk of bias, with several items classified as ‘high bias’ or ‘unclear bias.’ Only one study [26] classified ‘low bias’ for item 7 (‘Was the outcome assessor blinded?’) and all studies classified ‘high bias’ for item 9 (‘Are reports of the study free of selective outcome reporting?’).

**TABLE 3:** Risk of bias of in vitro studies included in the systematic review.

Author, year	Items of Golbach's tool							
	1	2	3	4	5	6	7	8
Ahmed et al. 2015 [20]	Unknown	Moderate	Unknown	Unknown	Unknown	Unknown	Unknown	Low
Ahmed et al. 2022 [21]	Unknown	Moderate	Unknown	Unknown	Unknown	Low	Unknown	Low
de Sousa et al. 2021 [22]	Unknown	Unknown	Unknown	Unknown	Unknown	Unknown	Unknown	Low
Osman et al. 2018 [23]	Unknown	Moderate	Unknown	Unknown	Unknown	Unknown	Unknown	Low
Radwan et al. 2008 [24]	Unknown	Moderate	Unknown	Unknown	Unknown	Unknown	Unknown	Low

*Note*: Item 1. Is a sham or dummy coil used for control treatment?; Item 2. Is the temperature controlled? Item 3. Was the exposure blinded? Item 4. Was the exposure randomised? Item 5. Is the cell vitality scored/measured? Item 6. Were the methods the same for control and exposure treatment? Item 7. Were the data measurements randomised? Item 8. Was there no industry sponsoring involved?

**TABLE 4:** Risk of bias of in vivo studies included in the systematic review.

Author, year	Items of SYRCLE's risk of bias									
	1	2	3	4	5	6	7	8	9	10
Akinola et al. 2018 [25]	Unclear	Unclear	Unclear	Unclear	Unclear	Unclear	Unclear	Unclear	High	Unclear
Campos et al. 2015 [26]	High	High	High	Unclear	Unclear	High	Low	Unclear	High	Unclear
Nwonuma et al. 2022 [27]	Unclear	Unclear	Unclear	Unclear	Unclear	Unclear	Unclear	Unclear	High	Unclear

*Note*: Item 1. Was the allocation sequence adequately generated and applied?; Item 2. Were the groups similar at baseline or were they adjusted for confounders in the analysis? Item 3. Was the allocation adequately concealed? Item 4. Were the animals randomly housed during the experiment? Item 5. Were the caregivers and/or investigators blinded from knowledge which intervention each animal received during the experiment? Item 6. Were animals selected at random for outcome assessment? Item 7. Was the outcome assessor blinded? Item 8. Were incomplete outcome data adequately addressed? Item 9. Are reports of the study free of selective outcome reporting? Item 10. Was the study apparently free of other problems that could result in high risk of bias?

## 4 Discussion

To the best of our knowledge, this is the first systematic review to summarise the evidence and assess the quality of the studies on the activity or toxicity of cannabis against *Plasmodium* sp. The findings suggest that cannabis and its derivatives exhibit activity against *P. falciparum* in vitro, with THC demonstrating high activity and CBD showing mild activity, indicating a potential antimalarial effect. Additionally, they have shown efficacy in reducing parasitemia and improving disease tolerance in animal models infected with *P. berghei* or *P. falciparum*. However, the toxicity of these compounds has not been thoroughly investigated. Moreover, most studies present an unknown or unclear risk of bias due to insufficient methodological details. Therefore, further well‐designed research is needed to confirm these findings.

This review identified a greater number of in vitro studies, which was expected, as these studies are less complex, more cost‐effective and easier to conduct than in vivo studies [28]. Future perspectives include the need to increase the number of in vivo studies to validate the promising results of the in vitro studies. Additionally, the development of more advanced experimental models, such as organoids and 3D culture systems, could help reduce the reliance on animal models [29].

Almost all in vitro studies were conducted in North America, specifically in the United States. All studies were funded by national research centers and drug abuse agencies, and the availability of research resources and infrastructure may help explain this. On the other hand, in vivo studies were mainly conducted in Africa and South America, where malaria is endemic [30, 31], which may explain the emphasis on animal models for testing antimalarial interventions. Conducting research in these regions is essential to ensuring that the findings apply to the populations most affected by the infection, particularly as they progress to human studies.

The cannabis plant comprises multiple species, with the three primary ones being *C. sativa* , *C. indica* and *C. ruderalis* [32]. Additionally, it contains various active compounds that can be classified as cannabinoids (e.g., THC and CBD) and non‐cannabinoids (e.g., terpenes and flavonoids) [32]. In the explored studies on malaria, *C. sativa* and their isolated cannabinoid compounds were the most studied. Although this review focused on cannabis‐derived compounds, other plant‐derived compounds exhibit antimalarial activity, such as machaeriol B isolated from *Machaerium multiflorum*, which demonstrated in vitro antimalarial activity against the *P. falciparum* W2 clone [33].

Studies demonstrated that THC [22, 23] presents high activity against *P. falciparum* and THC's mechanism of action does not appear to involve hemozoin formation inhibition, as it does for chloroquine [22]. Additionally, the included studies in this review reported the potential antimalarial effects of cannabis‐derived compounds through various mechanisms, primarily by modulating the immune response and directly inhibiting parasite growth. A previous study indicated a reduction of pro‐inflammatory cytokines by more than 90% in animal model tests [34]. Moreover, another study that performed a molecular docking analysis revealed that cannabis compounds effectively bind to *Plasmodium* sp. proteins, disrupting essential cellular functions [13]. The potential of cannabis‐derived compounds as antimalarial agents warrants further investigation to fully understand their mechanisms of action and therapeutic applicability.

There is a lack of further data on the toxicity of cannabis‐derived compounds in the included studies. Despite their therapeutic effects, their use may be associated with toxicities, particularly due to THC, including psychological events (anxiety, paranoia and psychosis), as well as neurological, gastrointestinal and cardiovascular disorders [35].

The assessment of the methodological quality of the studies revealed that, generally, the in vitro studies had an unknown risk of bias. Exceptions, such as temperature control and the absence of industry sponsorship, were classified as moderate and low risk, respectively. Industry funding of biomedical research can lead to biased study outcomes [36], making the absence of funding in these studies further strengthen the reliability of their results. On the other hand, the in vivo studies showed variability in risk of bias, with selective reporting of outcomes classified as high risk, and randomization, blinding of investigators and handling of incomplete data classified as unclear risk across all studies. These methodological limitations highlight the need for more rigorous standards in the planning of future studies to ensure the reliability and validity of the results.

This systematic review has several strengths, including the use of four databases and grey literature, which underscores the robustness of the comprehensive search. Moreover, the methodological quality of the studies ensured a thorough and rigorous reporting assessment of the reviewed literature. However, some limitations should be acknowledged. Articles were missed because they were not indexed in the databases searched or written in non‐Roman characters. Finally, the studies included in this review exhibited meaningful heterogeneity, which prevented a meta‐analysis.

Future research should prioritize well‐designed preclinical studies to clarify the mechanisms of action of cannabis‐derived compounds against Plasmodium sp. In addition, standardized methods for evaluating efficacy and toxicity are urgently needed, as current evidence remains limited and heterogeneous. Expanding investigations into diverse compound formulations, dosage regimens and their pharmacokinetic, pharmacodynamic and pharmacogenomic profiles may provide critical insights into therapeutic potential. Ultimately, rigorous studies addressing safety and long‐term effects are needed before these compounds can be considered as candidates for antimalarial therapy.

## 5 Conclusions

Cannabis and its derivatives have demonstrated mild activity for CBD compounds and high activity for THC compounds against *P. falciparum* and efficacy in reducing parasitemia and improving malaria disease tolerance in animal models, although they have not shown curative potential. However, the toxicity of these compounds has not been extensively studied. Additionally, most studies presented an unknown or unclear risk of bias due to a lack of detailed information in vitro studies and the absence of blinding and randomization in vivo studies.

Future research should provide more details on study design and confirm these findings, particularly regarding toxicity, to explore the safe and effective therapeutic use of cannabis.

## Ethics Statement

The authors have nothing to report.

## Conflicts of Interest

The authors declare no conflicts of interest.

## Supporting information


**Data S1:** tmi70044‐sup‐0001‐supinfo.docx.

## Data Availability

The datasets generated during and/or analyzed during this study can be obtained from the corresponding author on reasonable request.
