# Supplementary material for: Cannabis‐Derived Compounds Against Plasmodium sp.: A Systematic Review of Preclinical Studies
Source: Trop Med Int Health. 2025 Oct 15;31(1):1–9. doi: 10.1111/tmi.70044 (PMC12775889; doi:10.1111/tmi.70044)
Supplement: Supplementary file 1 — Data S1: tmi70044‐sup‐0001‐supinfo.docx. [file TMI-31-1-s001.docx]

**Supplementary material**

S1. Search strategy for all databases

| **Electronic Bibliographic Databases** | **Search Strategy** |
| --- | --- |
| Web of Science | #1 TS=(Cannabis OR Cannabi OR "Hemp Plant*" OR Marihuana OR Marijuana OR "Cannabis indica" OR Hemp* OR Ganja* OR Hashish* OR Bhang* OR "Cannabis sativa" OR "Medical Marijuana" OR "Medicinal Cannabis" OR "Marijuana Treatment" OR "Medicinal Marijuana" OR "Medical Cannabis" OR "Marijuana Dispensaries" OR "Cannabidiol" OR "Cannabinoids" OR "Cannabinol" OR "Dronabinol" OR "delta9-THC" OR "9-ene-Tetrahydrocannabinol" OR "9 ene Tetrahydrocannabinol" OR THC OR "delta1-Tetrahydrocannabinol" OR "delta1-THC" OR "delta9-Tetrahydrocannabinol" OR "Tetrahydrocannabinol" OR "Tetrahydrocannabinol, 6a-trans-Isomer" OR "Tetrahydrocannabinol, Trans-Isomer" OR "Tetrahydrocannabinol, Trans Isomer" OR "Tetrahydrocannabinol, 6aS-cis-Isomer" OR "Tetrahydrocannabinol, Trans-+--Isomer" OR Marinol OR "Tetrahydrocannabinol, 6aR-cis-Isomer" OR CBD OR Phytocannabinoid*)  #2 TS=("Plasmodium" OR "Malaria" OR "Plasmodium Infection*" OR "Remittent Fever" OR "Marsh Fever" OR "Paludism")  #3 #1 AND #2 |
| Medline (Pubmed) | #1 "Cannabis"[Mesh] OR Cannabis OR Cannabi OR "Hemp Plant*" OR Marihuana OR Marijuana OR "Cannabis indica" OR Hemp* OR Ganja* OR Hashish* OR Bhang* OR "Cannabis sativa" OR "Medical Marijuana"[Mesh] OR "Medical Marijuana" OR "Medicinal Cannabis" OR "Marijuana Treatment" OR "Medicinal Marijuana" OR "Medical Cannabis" OR "Marijuana Dispensaries" OR "Cannabidiol"[Mesh] OR Cannabidiol OR "Cannabinoids"[Mesh] OR Cannabinoids OR "Cannabinol"[Mesh] OR Cannabinol OR "Dronabinol"[Mesh] OR Dronabinol OR delta9-THC OR 9-ene-Tetrahydrocannabinol OR 9 ene Tetrahydrocannabinol OR THC OR delta1-Tetrahydrocannabinol OR delta1-THC OR delta9-Tetrahydrocannabinol OR Tetrahydrocannabinol OR Tetrahydrocannabinol, 6a-trans-Isomer OR Tetrahydrocannabinol, Trans-Isomer OR Tetrahydrocannabinol, Trans Isomer OR Tetrahydrocannabinol, 6aS-cis-Isomer OR Tetrahydrocannabinol, Trans-+--Isomer OR Marinol OR Tetrahydrocannabinol, 6aR-cis-Isomer OR CBD OR Phytocannabinoid*  #2 "Plasmodium"[Mesh] OR "Plasmodium" OR "Plasmodium vivax"[Mesh] OR "Plasmodium vivax*" OR "Plasmodium falciparum"[Mesh] OR "Plasmodium falciparum*" OR "Malaria"[Mesh] OR "Malaria" OR "Plasmodium Infection*" OR "Remittent Fever" OR "Marsh Fever" OR "Paludism"  #3 #1 AND #2  #4 "review"[Publication Type] OR "scientific integrity review"[Publication Type] OR "systematic review"[Publication Type] OR "meta analysis"[Publication Type] OR "guideline"[Publication Type] OR "congress"[Publication Type] OR "letter"[Publication Type] OR "book illustrations"[Publication Type] OR "clinical conference"[Publication Type] OR "clinical trial protocol"[Publication Type] OR "editorial"[Publication Type] OR "video audio media"[Publication Type]  #5 #3 NOT #4 |
| Scopus | TITLE-ABS-KEY(Cannabis OR Cannabi OR "Hemp Plant*" OR Marihuana OR Marijuana OR "Cannabis indica" OR Hemp* OR Ganja* OR Hashish* OR Bhang* OR "Cannabis sativa" OR "Medical Marijuana" OR "Medicinal Cannabis" OR "Marijuana Treatment" OR "Medicinal Marijuana" OR "Medical Cannabis" OR "Marijuana Dispensaries" OR "Cannabidiol" OR "Cannabinoids" OR "Cannabinol" OR "Dronabinol" OR "delta9-THC" OR "9-ene-Tetrahydrocannabinol" OR "9 ene Tetrahydrocannabinol" OR THC OR "delta1-Tetrahydrocannabinol" OR "delta1-THC" OR "delta9-Tetrahydrocannabinol" OR "Tetrahydrocannabinol" OR "Tetrahydrocannabinol, 6a-trans-Isomer" OR "Tetrahydrocannabinol, Trans-Isomer" OR "Tetrahydrocannabinol, Trans Isomer" OR "Tetrahydrocannabinol, 6aS-cis-Isomer" OR "Tetrahydrocannabinol, Trans-+--Isomer" OR Marinol OR "Tetrahydrocannabinol, 6aR-cis-Isomer" OR CBD OR Phytocannabinoid*) AND TITLE-ABS-KEY("Plasmodium" OR "Malaria" OR "Plasmodium Infection*" OR "Remittent Fever" OR "Marsh Fever" OR "Paludism") AND NOT DOCTYPE("ab" OR "bk" OR "ch" OR "bz" OR "cp" OR "cr" OR "dp" OR "ed" OR "er" OR "le" OR "mm" OR "no" OR "pr" OR "tb" OR "re" OR "sh") OR SRCTYPE("b" OR "k" OR "p" OR "m" OR "n" OR "w" OR "l") |
| Lilacs | MH:Cannabis OR Cannabis OR Cannabi OR "Hemp Plant*" OR Marihuana OR Marijuana OR "Cannabis indica" OR Hemp* OR Ganja* OR Hashish* OR Bhang* OR "Cannabis sativa" OR "Medical Marijuana" OR "Medicinal Cannabis" OR "Marijuana Treatment" OR "Medicinal Marijuana" OR "Medical Cannabis" OR "Marijuana Dispensaries" OR "Cannabidiol" OR "Cannabinoids" OR "Cannabinol" OR "Dronabinol" OR "delta9-THC" OR "9-ene-Tetrahydrocannabinol" OR "9 ene Tetrahydrocannabinol" OR THC OR "delta1-Tetrahydrocannabinol" OR "delta1-THC" OR "delta9-Tetrahydrocannabinol" OR "Tetrahydrocannabinol" OR "Tetrahydrocannabinol, 6a-trans-Isomer" OR "Tetrahydrocannabinol, Trans-Isomer" OR "Tetrahydrocannabinol, Trans Isomer" OR "Tetrahydrocannabinol, 6aS-cis-Isomer" OR "Tetrahydrocannabinol, Trans-+--Isomer" OR Marinol OR "Tetrahydrocannabinol, 6aR-cis-Isomer" OR CBD OR Phytocannabinoid* AND MH:Plasmodium OR Plasmodium OR MH:Malaria OR Malaria OR "Plasmodium Infection*" OR "Remittent Fever" OR "Marsh Fever" OR "Paludism" OR MH:"Malaria, Cerebral" AND NOT type_of_study:"systematic_reviews" OR "sysrev_observational_studies" OR "overview" |
| Google Scholar | (Cannabis OR "Medical Marijuana" OR Cannabinoids OR Cannabinol OR Dronabinol OR THC OR CBD) AND (Plasmodium OR Malaria)  Exclude: patents and citations |
